# Influential Factors of Residents’ Health in Regions of China: A Comparative Study

**Published:** 2018-08

**Authors:** Zhiguang ZHANG, Haiqing HU, Conghui HAO, Kaikai ZHANG

**Affiliations:** 1. Dept. of Business Administration, Xi’an University of Technology, Xi’an, China; 2. Dept. of Accounting and Finance, Xi’an University of Technology, Xi’an, China; 3. Dept. of Economy and Trade, Xi’an University of Technology, Xi’an, China; 4. Dept. of Student Affairs, Shaanxi University of Science and Technology, Xi’an, China

**Keywords:** Human mortality, Environmental pollution, Economic growth, Panel regression model

## Abstract

**Background:**

With the rapid growth of China’s economy, the country’s economic, social, and environmental development has also shown significant differences in various regions. Improving the health level of residents has gradually become a hot issue. We aimed to explore the difference of the health level of the residents in different regions of China and its influential factors.

**Methods:**

A panel regression model was established using statistical data from 31 provinces in the eastern, central, and western regions of China from the years 2000 and 2016 to examine the influence of economic, environmental, and social factors on the health level of residents in these regions and investigate the regulating role of public service factors.

**Results:**

Environmental pollution significantly promotes human mortality in the eastern and central regions of China. The urbanization rate significantly reduces human mortality in the eastern and central regions. Per capita gross domestic product in the eastern and western regions has a significant U-shaped relationship with human mortality. The number of beds in health institutions per 10,000 persons, average years of schooling, and endowment insurance contribution service have varying degrees of influence on residents’ health level in the three regions of China. In particular, endowment insurance contribution service significantly reduces human mortality in the eastern region, whereas its impact on the western region is insignificant.

**Conclusion:**

Differential policies in environmental pollution control, economic growth, and medical and public health should be formulated to reduce mortality in China, specifically in the eastern, central, and western regions.

## Introduction

**Fig. 1:**
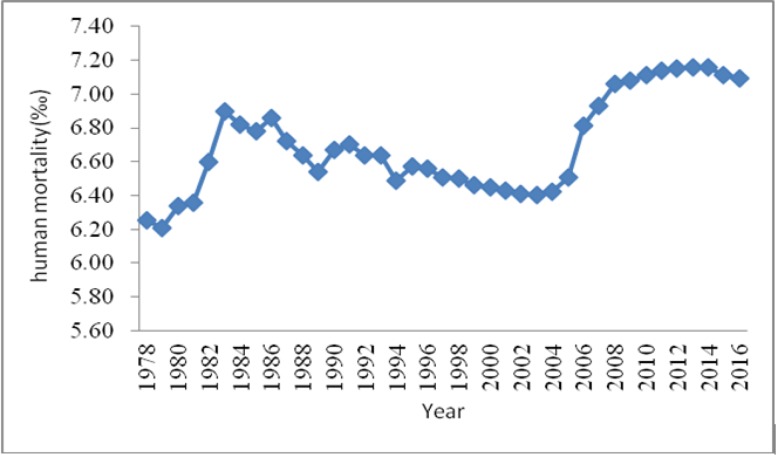
Human mortality in China since reform and opening up

Since the reform and opening up in 1978, the level of China’s economic and social development has achieved great progress. Per capita gross domestic product (GDP) increased from 223.84 US dollars in 1978 to 8126.70 US dollars in 2016, and the per capita disposable income of urban residents increased from 199.42 US dollars to 5060.90 US dollars (1). Thus, the population’s living standards have been significantly elevated. However, compared with the achievement at the economic level, improvement at the residents’ health level is insignificant. Fig. 1 shows the trend of human mortality in China from 1978 to 2016 (1–2). This trend indicates that improvement in the health level of the residents cannot rely only on economic growth and enhanced medical and health conditions.

This study shifted the focus to various social and environmental factors and public services that affect residents’ health to explore the decisive factors of the health level of residents. By doing so, we aimed to reasonably explain the trend of human mortality in China and provide reference for medical care and environmental and social policies to promote improvement in residents’ health.

In recent years, the factors that affect the residents’ health in China have grown in number and complexity. In addition to previous economic factors and medical and health conditions, several new influential factors have exerted an important impact on the residents’ health. The worsening environmental pollution gravely endangered the health of residents, and incidence of environmentally related diseases significantly increased (3). In addition, the acceleration of China’s urbanization has increased the health risks of landless farmers and damaged the health of rural residents (4). The health problems brought about by the aging of the population have also become a concern for the government and scholars.

Thus, determining the main factors that affect health and the degrees of influence of these factors is key to improving the residents’ health. Importantly, regional differences in the health of Chinese residents are expanding due to the imbalance between economic and social development in various regions in China (5). Appropriate understanding of these differences in residents’ health is of great significance for the elimination of health inequalities among regions and improvement of China’s medical and health system. We examined the influence of economic, environmental, and social factors on residents’ health among regions using the panel regression model and analyzed the mediating role of public services on the basis of statistical data from 31 provinces in the eastern, central, and western regions of China.

### Literature review

Scholars closely followed the studies on these factors for a long time. Etchie et al. (6) evaluated PM2.5 concentration in nine regions in India in 2013 and confirmed that air pollution caused major losses to the residents’ health, and its influence on health would exist extensively. Lima et al. (7) conducted a study using the random effect regression model with the health data of 18,484 elderly persons in Brazil in 2008 and found that their health was influenced by not only personal income but also differences in regional incomes.

Chen et al. (8) studied the relationship between air pollution and respiratory system diseases of residents in four cities in northern China, and the results showed that an increase in PM10 by 10 μg/m^3^ increased mortality from respiratory system diseases by 24%. Wu et al. (9) found that the monthly income and daily working time had a significant effect on the health of urban residents. Cheng et al. (10) empirically analyzed the relationship between urbanization and residents’ health in China in 2005 and 2011 and found that increased urbanization rate could prolong life expectancy and reduce infant mortality. On the contrary, Some researches suggested that the higher the level of urbanization, the lower the health level of the residents, because the negative effects of urbanization on health exceeded the positive effects, such as increase in income, improvement of nutrition, and enhancement of medical facilities (11). Wang et al. (12) introduced the proportion of female residents into the model as a social variable on the basis of the Grossman health production function; results showed that an increase in female proportion led to a decrease in human mortality.

Cao et al. (13) studied the influence of public health expenditure on the health of the human capital in China in 2003 and 2013; results indicated that public health expenditure as a percentage of GDP reached over 0.81%, which applied a positive effect on the health of the human capital. Sun et al. (14) studied the relationship between health input and residents’ health in China and found that an increase by 1% in health input at the grassroots level promoted a decrease in the health of the residents by 0.142%. Regidor et al. (15) studied the relationship between education and human mortality from ages 25 to 74 years in Madrid, Barcelona, and Basque, Spain and concluded that educational inequality had a critical impact on mortality. Lastly, Gao (16) studied the matching problem between pension insurance mechanism and public health policy and found that pension insurance mechanism and life expectancy had a two-way adjustment effect. The above mentioned studies showed that several factors affect the health level of residents.

For the research on the regional, urban, and rural differences in the health level of residents, Hong et al. (17) found that social and economic inequality intensified health inequity among regions in South Korea; increased equitable income distribution and enhanced average income were required to reduce health inequality. Du et al. (18) analyzed the health conditions of the elderly in time and space with data from the Chinese Longitudinal Healthy Longevity Survey and found that the health inequality among the rural elderly was worse than that among the urban elderly. Zhai et al. (19) studied the difference in health security of 1,198 urban and rural residents in five cities in China and revealed a highly significant difference with that in the medical service as the main factor that affected the degree of health security between urban and rural residents.

We aimed to determine the influencing factors of Chinese residents’ health and explore the comprehensive influence of these factors on the residents’ health in different regions of China. This study mainly differs from previous research in the following points: 1. studying the comprehensive effects of economic, environmental, social, and public service factors on residents’ health and 2. studying the differences in the degree of influence of these factors on residents’ health in the eastern, central, and western regions in China.

## Methods

### Variable selection and data source

The demand for health funds is dependent on the benefits and costs of health (20). The main factors that affect the demand for health funds cover age, education, and income. Therefore, improving health is closely related to many factors rather than a single one. Behrman et al. (21) posited that all countries should select various input factors that affect health on the basis of their conditions rather than blindly learning from or copying the theoretical models of developed countries. On the basis of this notion, the variables selected for this study are derived according to the actual situation in China and are presented as follows:

#### Health Level of Residents

According to the definition given and empirical study conducted by the WHO, health is largely measured by life expectancy and human mortality in related research (12). Human mortality can holistically measure and reflect the health status of a country or region. Considering the availability of data, a variable for human mortality, namely, ln *swl_jt_* is used to measure the health level of the residents in this study.

#### Environmental factors

Compared with other environmental pollution elements, the spillover effects of the air environment are stronger. The residents are more frequently exposed to the atmosphere than to other environmental factors (water and soil). This scenario can well reflect the dose–response relationship between pollutants and health at the macro scale. *SO*_2_ is the main source of fine particulate matter (PM2.5). Hence, several scholars prefer its use as an environmental index (22). Therefore, the variable for *SO*_2_ emissions, that is, ln *s_jt_*, is selected to measure environmental factors.

#### Economic factors

Economic growth can increase the supply quantity of health facilities, provide good social security for residents, and thus effectively improve life expectancy and health conditions. The measurement index for economic growth is relatively mature. It is often expressed as per capita GDP, and has an inverted U-shaped relationship with health (23). For the sake of stability, this study introduced per capita GDP (ln *agdp_jt_*) and the square of the per capita GDP (ln^2^ *agdp_jt_*) to investigate the influence of economic factors on the health level of residents.

#### Social factors

The social environment of the residents also has an important impact on residents’ health. This study adopted the variables of urbanization rate (ln *urban_jt_*), ratio of the female population (ln *fema_jt_*), and population of the elderly (ln *old_jt_*) to represent social factors. Urbanization can provide residents enhanced health care and living conditions. Simultaneously, the rapid pace of life caused by urbanization can lead to a decline in health. In addition, women’s health level is considered different from that of men from the perspective of genetics due to the differences in physiological structure. Therefore, the proportion of women in the population can also affect the health level of the residents. According to a WHO forecast, the group of population aged 60 years and above will account for approximately 35% of the total population by 2050, which will have a massive impact on residents’ health.

#### Pubic services

Medical and health, educational, social security, and other public services that a country or region can provide also affect the health of residents. This study selected the number of beds in health institutions per 10,000 persons (ln *abed_jt_*), average years of schooling (ln *edu_jt_*), and number of endowment insurance contributors (ln *ei_jt_*) to measure the influence of China’s public services and education and social security on residents’ health. For the above mentioned variables, ln(*) represents taking the natural logarithm to eliminate the influence of homoskedasticity. The study selects data from 31 provinces and cities in China (except Hong Kong, Macao, and Taiwan) from the period of 2000 to 2016 as sample. The data are mainly derived from such sources as the *China Statistical Yearbook*, *China Environmental Statistics Yearbook*, *China Health Statistics Yearbook*, and other statistical yearbooks of various provinces and cities in China. The eastern, central, and western regions in China are divided as follows. The eastern region covers Beijing, Tianjin, Hebei, Liaoning, Shanghai, Jiangsu, Zhejiang, Fujian, Shandong, Guangdong, and Hainan. The central region is made up of Shanxi, Inner Mongolia, Jilin, Heilongjiang, Anhui, Jiangxi, Henan, Hubei, and Hunan. Lastly, the western region comprises Guangxi, Chongqing, Sichuan, Guizhou, Yunnan, Tibet, Shaanxi, Gansu, Qinghai, Ningxia, and Xinjiang.

#### Research methods

According to the actual situation of China, the environmental factors and social factors are introduced to the macro health production function established by Zhao’ research (24). This study aimed to observe the influence of the environmental problem and urbanization in China with a large concern on the residents’ health. Fig. 1 reflects that human mortality in China shows a time trend. Thus, this study introduced the time variable *t* to reflect the changing trend of the selected samples according to region and time. To reduce the influence of multiple collinearity, panel regression model 1 is established as follows:

Model 1:

$$\text{ln}\;{\mathrm{swl}}_{\mathrm{jt}}=b_{0}{\mathrm{lnagdp}}_{\mathrm{jt}}+b_{2}{\ln2\mathrm{agdp}}_{\mathrm{jt}}+b_{3}{\mathrm{lns}}_{\mathrm{jt}}+b_{4}{\mathrm{lnurban}}_{\mathrm{jt}}+b_{5}{\mathrm{lnfema}}_{\mathrm{jt}}+b_{6}{\mathrm{old}}_{\mathrm{jt}}+b_{7}t+w_{\mathrm{jt}}$$

where ln *swl_jt_* represents human mortality and is used to represent the health level of residents in the study. ln *agdp_jt_* and ln^2^ *agdp_jt_* refer to per capita GDP and its square, respectively, and represent the economic factors. ln *s_jt_* refers to *SO*_2_ emissions and represents environmental factors. ln *urban_jt_*, ln *fema_jt_*, and ln *old_jt_* refer to the urbanization rate, ratio of the female population, and population of the elderly. *b*_0_ is a constant term, and *b*_1_ and *b*_2_ to *b*_6_ refer to the elastic coefficients corresponding to different variables. *w_jt_* is a random disturbance term. To reflect the moderating effect of public services on the impacts of economic, environmental, and social factors on the health level of residents, the study constructs panel regression model 2 as follows:

Model 2:

$$\text{ln}\;{\mathrm{swl}}_{\mathrm{jt}}=b_{0}{\mathrm{lnagdp}}_{\mathrm{jt}}+b_{2}{\ln2\mathrm{agdp}}_{\mathrm{jt}}+b_{3}{\mathrm{lns}}_{\mathrm{jt}}+b_{4}{\mathrm{lnurban}}_{\mathrm{jt}}+b_{5}{\mathrm{lnfema}}_{\mathrm{jt}}+b_{6}{\mathrm{old}}_{\mathrm{jt}}+b_{7}t+b_{8}{\mathrm{lnabed}}_{\mathrm{jt}}+b_{9}{\mathrm{lnedu}}_{\mathrm{jt}}+b_{10}{\mathrm{lnei}}_{\mathrm{jt}}+w_{\mathrm{jt}}$$

where ln *abed_jt_*, ln *edu_jt_*, and ln *ei_jt_* refer to the number of beds in health institutions per 10,000 persons, average years of schooling, and number of endowment insurance contributors, respectively. These variables represent public services.

## Results

### Unit root test

To ensure the robustness of the regression results, a unit root test must be performed before the regression analysis of panel data. This study selected two methods of the unit root test. These methods were 1) LLC statistics with the cross-section sequence containing the unit root and 2) PP statistics with the cross-section sequence without the unit root. Table 1 presents the test results of the variables using the two unit root test methods under three assumptions: with only the intercepting term, with the intercepting and trend terms, and without the intercepting and trend terms. Results show that the two test methods pass the LLC and PP tests under all assumptions. Therefore, the panel data are considered stationary.

**Table 1:** Panel Unit Root Test

***Variable***	***With Intercepting Term***		***With Intercepting and Trend***		***Without Intercepting and Trend***	
	***LLC Same Root Hypothesis***	***PP Different Root Hypothesis***	***LLC Terms Same Root Hypothesis***	***PP Different Root Hypothesis***	***LLC Terms Same Root Hypothesis***	***PP Different Root Hypothesis***
ln *swl_jt_*	−27.599(0.00)	686.577(0.00)	−22.654(0.00)	570.601(0.00)	−36.712(0.00)	628.634(0.00)
ln *agdp_jt_*	−19.636(0.00)	338.528(0.00)	−21.763(0.00)	531.674(0.00)	−22.838(0.00)	519.943(0.00)
ln^2^ *agdp_jt_*	−19.319(0.00)	331.207(0.00)	−22.959(0.00)	530.471(0.00)	−22.738(0.014)	519.497(0.74)
ln *s_jt_*	−8.579(0.00)	226.547(0.00)	−8.034(0.00)	188.259(0.00)	−20.097(0.00)	354.768(0.00)
ln *urban_jt_*	−28.177(0.00)	653.564(0.00)	−26.880(0.00)	534.131(0.00)	−31.218(0.00)	632.686(0.00)
ln *fema_jt_*	−18.952(0.00)	717.711(0.00)	−11.578(0.00)	600.408(0.00)	−29.302(0.00)	629.304(0.00)
ln *old_jt_*	−25.345(0.00)	681.251(0.00)	−19.028(0.00)	592.009(0.00)	−31.064(0.00)	675.795(0.00)
ln *abed_jt_*	−26.188(0.00)	453.732(0.00)	−22.116(0.00)	499.447(0.00)	−29.225(0.00)	598.337(0.00)
ln *edu_jt_*	−23.350(0.00)	693.553(0.04)	−19.644(0.00)	571.446(0.00)	−30.713(0.026)	636.240(0.00)
ln *ei_jt_*	−24.771(0.00)	408.782(0.00)	−17.905(0.00)	396.536(0.00)	−28.797(0.00)	497.821(0.00)

### Co-integration test and model discrimination

The above mentioned variable sequences are integrated of order 2, which conforms to the precondition of the co-integration test. We adopted the Kao test method for the co-integration test because many explanatory variables were acting on the model. The Kao test includes the DF and ADF tests. In this study, the ADF statistics were selected. The original hypothesis of the test is that a co-integration relationship does not exist among the variables. According to the results of the co-integration test, the ADF statistics of the country and the eastern, middle, and western regions are −6.670, −4.386, −4.412, and −4.933, respectively, which pass the significance test of 1%. Hence, the original hypothesis is rejected, and a co-integration relationship exists between the explanatory and explained variables in each region.

In addition, the Hausman test is conducted before establishing the panel data model. Results show that the random effect model is suitable for this study.

### Regression analysis of the panel data model

We established the panel data model with human mortality as the independent variable and the influencing factors as the dependent variables, according to Models 1 and 2. Panel regression is performed on the health level of residents in the country and in the eastern, central, and western regions. Table 2 provides the statistical results.

According to Table 2, whether or not the impact of public services is considered, *SO*_2_ emissions play a significant role in promoting human mortality in the eastern and central regions in China. In Model 1, for example, the regression coefficients of *SO*_2_ emissions to human mortality in the eastern and central regions are 0.026 and 0.038, respectively, which are significant at the 5% level. In Model 2, the regression coefficients of *SO*_2_ emissions to human mortality in the eastern and central regions are 0.053 and 0.046, respectively, which are significant at the 1% level. The regression coefficient of *SO*_2_ emissions to human mortality in the western region is −0.034 in Model 1 and positive but insignificant under Model 2.

**Table 2:** Results of panel regression analysis for influential factors of residents’ health (Model 1 & Model 2)

***Variable***	***China***		***Eastern Region***		***Central Region***		***Western Region***	
	***Model 1***	***Model 2***	***Model 1***	***Model 2***	***Model 1***	***Model 2***	***Model 1***	***Model 2***
ln *agdp_jt_*	−0.235^***^ (0.007)	−0.438^***^ (0.000)	0.075 (0.784)	−0.489^*^ (0.079)	−0.259 (0.332)	−0.173 (0.548)	−0.396^**^ (0.011)	−0.824^***^ (0.000)
ln^2^ *agdp_jt_*	0.011^**^ (0.013)	0.020^***^ (0.000)	−0.004 (0.745)	0.025^**^ (0.048)	0.008 (0.546)	0.004 (0.763)	0.026^***^ (0.001)	0.033^***^ (0.008)
ln *s_jt_*	−0.005 (0.455)	0.021^***^ (0.004)	0.026^**^ (0.028)	0.053^***^ (0.000)	0.038^**^ (0.020)	0.046^***^ (0.001)	−0.034^***^ (0.000)	0.018 (0.170)
ln *urban_jt_*	0.005 (0.839)	0.011 (0.601)	−0.086 (0.161)	−0.181^***^ (0.001)	−0.096 (0.151)	−0.142^**^ (0.047)	−0.006 (0.824)	0.001 (0.986)
ln *fema_jt_*	0.132 (0.473)	0.176 (0.290)	0.174 (0.489)	0.645^**^ (0.015)	−0.369 (0.492)	−1.065^**^ (0.041)	−0.349 (0.275)	−0.832^*^ (0.059)
ln *old_jt_*	0.194^***^ (0.000)	0.216^***^ (0.000)	0.223^***^ (0.000)	0.323^***^ (0.000)	0.155^***^ (0.006)	0.192^***^ (0.000)	0.139^***^ (0.002)	0.434^***^ (0.000)
ln *abed_jt_*		0.156^***^ (0.000)		0.122^***^ (0.000)		0.132^***^ (0.005)		0.218^***^ (0.000)
ln *edu_jt_*		−0.363^***^ (0.000)		−0.491^***^ (0.000)		−0.094 (0.477)		−0.436^***^ (0.000)
ln *ei_jt_*		−0.003 (0.820)		−0.094^***^ (0.000)		0.077^***^ (0.000)		0.001 (0.960)
*t*	−0.003 (0.163)	−0.003 (0.152)	−0.000 (0.912)	0.006^*^ (0.059)	0.018^***^ (0.001)	0.007 (0.201)	−0.022^***^ (0.000)	0.008 (0.106)
Constant	2.110^***^ (0.006)	3.101^***^ (0.000)	0.499 (0.759)	2.611 (0.140)	4.737^**^ (0.045)	6.345^***^ (0.005)	4.579^***^ (0.001)	8.938^***^ (0.000)
Sample Size	525	522	187	184	152	152	186	186

* *p* < 0.05,

** *p* < 0.01,

*** *p* < 0.001.

Two-tailed test; Standard errors are shown in parentheses.

This result indicates that the influence of *SO*_2_ emissions on human mortality in the western region is insignificant. Thus, the harm brought about by *SO*_2_ emissions to the health level of residents in the eastern and central regions is greater than that in the western region. It is closely related to the level of *SO*_2_ emissions. In addition, a comparison of the regression coefficients of the three major regions under Models 1 and 2 shows that the regression coefficient of *SO*_2_ emissions increases after the introduction of public services. This finding indicates that the current public services, such as medical, cannot effectively reduce the effect of *SO*_2_ emissions on the residents’ health.

In terms of the social variables, the regression coefficients of urbanization rate to human mortality in China and the three regions are insignificant when the regulating effect of public services is not considered. However, the opposite is true when the regulating effect of public services is considered. That is, the regression coefficients of urbanization rates in the eastern and central regions are −0.181 and −0.142, which are significant at the 1% and 5% significance levels, respectively. This result indicates that urbanization in the eastern and central regions provides additional medical and health institutions, enhanced educational services, and new endowment insurance services, thereby reducing mortality and increasing the health level of residents.

After introducing public services in the western region, urbanization does not reduce human mortality. This notion indicates that urbanization in the western region seriously lags behind those in the eastern and central regions. Therefore, the western region’s current urbanization cannot provide improved public services for the residents.

The regression coefficients of the female population ratio to human mortality in China and the three regions are insignificant without considering the regulating effect of public services. After introducing this regulation, the regression coefficient of the female population ratio in the eastern region becomes 0.645 at the 5% significance level. This finding indicates that the medical and health services, education, and endowment insurance provided for women in the eastern region cannot reduce the mortality of women because career women in the well-developed eastern region are faced with more occupational pressure than those in the central and western regions. Meanwhile, the scenarios in the central and western regions are opposite. The regression coefficients of the female population ratio are −1.065 and −0.832, respectively, which are both significant. This notion indicates that public services in the central and western regions can effectively reduce the mortality of women and, in turn, reduce human mortality.

The effect coefficients of population aging are positive in the eastern, central, and western regions, which meets our expectations. The higher the aging of the society, the worse the residents’ health (25–26). According to the estimated results under Model 2, the regression coefficients of the degree of aging in China and the three regions are 0.216, 0.323, 0.192, and 0.434, respectively, which are significant at the 1% level. By contrast, public services in the western region seriously aggravate the harm brought about by aging to the health of the residents. Thus, the western region should provide appropriate medical and health care services, education, and endowment insurance for the elderly.

At this juncture, the influence of public services on the health of residents is analyzed in detail. The regression coefficients of the number of beds in health institutions per 10,000 persons are positive in China and the three regions at 0.156, 0.122, 0.132, and 0.218, which are significant at the 1% level. This result indicates that the existing number of beds in health institutions cannot effectively reduce human mortality. Thus, the effect of hospitalization on health improvement is limited, and the waste of public health resources has a negative effect on health. This notion is in agreement with the conclusions obtained by Wang J et al. (12). It also reflects the low level of efficiency of China’s medical system, especially in the western region. The effect of the average years of schooling on residents’ health is evident and statistically significant in the eastern and western regions, which is consistent with the theoretical analysis of Grossman. Education not only increases the residents’ knowledge of prevention and health care but also promotes a healthy lifestyle. The average number of schooling years in the western region plays an important role in reducing human mortality. Therefore, improving the level of education is vital to improving the health of residents. The influence of the number of endowment insurance contributors on the health of residents varies according to region; the coefficient is significantly negative in the eastern region and significantly positive in the central region. As China has an aging society, the influence of the health level of the elderly on the health of the overall society cannot be overlooked. Endowment insurance reduces the prices of public services through the compensation or sharing mechanism such that the elderly can take advantage of public services. In this manner, human mortality is reduced, as confirmed in the eastern region. In the central region, the increase in the number of endowment insurance contributors cannot improve public health. Thus, the social security policy in the central region needs improvement.

The influence of the economic factors on public health is further examined. From the panel regression estimation results, the regression coefficients of the economic factors mostly pass the test at the 10% significance level. The first-order coefficients of per capita GDP in China and the eastern and central regions are negative, whereas the second-order coefficients are positive. This result indicates that a U-shaped relationship exists between human mortality and economic growth in these regions. The increase in per capita GDP makes improved living conditions and medical services possible for the residents and promotes an increase in the health investment of residents, thereby reducing human mortality. However, with the growth of per capita GDP, human mortality does not consistently decrease over time, although it has an inflection point. Such a case indicates that when economic development reaches a certain level, it will cease to be a decisive factor of residents’ health. Instead, living environment, work stress, physical exercise, and good lifestyle become important (27–28).

## Discussion

Results show that the influence of environmental pollution on the health level of residents varies according to region. *SO*_2_ emissions in the eastern and central regions are evidently higher than those in the western region, and population density in the eastern region is remarkably higher than that in the western region (29). Therefore, controlling environmental pollution in the economically developed eastern and central regions is necessary to realize the coordinated development between economy and environment.

We also found that a U-shaped relationship existed between human mortality and economic growth in China and the eastern and western regions. In the early period of economic growth, per capita household food expenditure and quality of life increases and health of residents improves. However, with the continuous improvement of the quality of life, their understanding of health care and concept of a healthy life have not synchronously improved, thus leading to chronic diseases, such as diabetes and hypertension (30). Therefore, with the continued increase in per capita GDP, improving the lifestyle of the residents and creating a good living environment for the improvement of the health level of residents in the eastern region are of significance.

Moreover, we found that the urbanization process did not necessarily improve the health level of residents. This conclusion supports Wang’s view of the low synergy between the current urbanization level and residents’ health in China (31). Attention should be paid to the provision of supporting medical, education, and endowment insurance services as part of the urbanization of the western region.

The number of endowment insurance contributors significantly promotes residents’ health in the eastern region, whereas this increase in the central region does not improve public health. Thus, the social security policy in the central region requires improvement. Furthermore, China’s endowment insurance service should be inclined to the poorly developed areas in the central and western regions.

In view of the problems identified in the study, the following solutions are proposed. First, formulating appropriate policies for environmental pollution control and economic development in different regions is necessary in achieving coordinated development between the economy and environment and reducing the health risks caused by environmental pollution. Second, attention should be paid to the supporting medical, education, and endowment insurance services provided during urbanization. Third, medical and health policies should emphasize not only the quantity indexes, such as investment in public health and training of health technicians, but also further improvement of the operating efficiency of the health system and quality of public medical services. Doing so will reduce the health deterioration of residents caused by the poor efficiency of medical services.

## Conclusion

On the basis of provincial panel data of China from the years 2000 and 2016, the study established a random effect model to explore the influencing factors that affect the health level of residents in the three major economic regions in China. Results show that economic, environmental, social, and public services factors have varying degrees of influence on residents’ health in the three major economic regions. China should formulate differential policies in environmental pollution control and economic growth according to region, pay attention to the improvement in the quality and efficiency of public services, and actively and steadily promote the health development of Chinese residents.

## Ethical considerations

Ethical issues (Including plagiarism, Informed Consent, misconduct, data fabrication and/or falsification, double publication and/or submission, redundancy, etc.) have been completely observed by the authors.
